# Trigeminal Trophic Syndrome: Report of 2 Cases

**Published:** 2013-11-15

**Authors:** Yoko Osaki, Tateki Kubo, Kyosuke Minami, Daisuke Maeda

**Affiliations:** Department of Plastic Surgery, Osaka Rosai Hospital, Sakai, Japan

**Keywords:** nasal ala, nasal reconstruction, trigeminal nerve, trigeminal trophic syndrome, ulceration

## Abstract

**Objective:** We present 2 cases of trigeminal trophic syndrome treated by surgery. **Methods:** We performed reconstruction of the ala nasi using a nasolabial flap or paramedian forehead flap in combination with an auricular chondrocutaneous composite graft. **Results:** One case was successfully treated. However, ulceration recurred intermittently in the other case. **Conclusions:** Although trigeminal trophic syndrome is rare, we believe that plastic surgeons should have a raised awareness of this entity and familiarity with the treatment options.

Trigeminal trophic syndrome (TTS) is a rare condition deriving from damage to the trigeminal nerve system. Patients of TTS develop skin ulcers in the trigeminal sensory region, most frequently at the nasal ala because of self-manipulation of the lesion in response to an altered sensation. The classic clinical appearance of TTS involves 1 or more crescent-shaped lateral nasal ala ulcerations. Differential diagnosis with other entities, such as skin neoplasms, vasculitis, infections, and granulomatous disease, is necessary. Furthermore, a positive neurologic history of the trigeminal system, such as stroke, treatment of trigeminal neuralgia, brain tumor, and postencephalopathic Parkinsonism, also is of importance. Recurrence of skin ulcers in TTS patients is common, and many therapeutic options have been reported, including behavioral modification, pharmaceutical intervention, installation of a protector, transcutaneous nerve stimulation, and surgical repair. Here, we report 2 patients of TTS treated by surgery.

## CASE REPORTS

### Case 1

**Figure 1:**
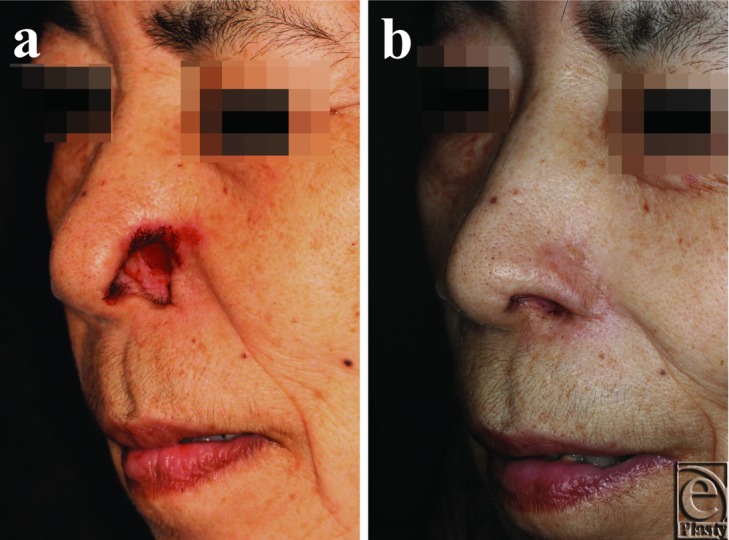
Case 1. (*a*) Preoperative view of the patient at the time of presentation. (*b*) Postoperative view at 30 months from the first operation.

A 62-year-old woman was referred to us with ulceration of her left nasal ala, and xeransis of the nasal mucosa (Fig 1a). She had undergone brain tumor excision 4 times in the past 10 years, which led to damage from the third to sixth cranial nerves and decreased sensation in the left side of her face. Approximately 2 years before visiting us, she became conscious of a crust on her nose and developed an injury from scratching the area. She described numbness of the left face, and her family described her unconscious self-manipulation of the nose. The cause of the alar ulceration was first investigated by biopsy. The biopsy result revealed a nonspecific chronic inflammation without evidence of neoplasm and vasculitis. Autoimmune process was also excluded by immunohistochemistry. The results of laboratory tests, including a complete blood count, basic chemistry, antinuclear antibody, rheumatoid factor, cytoplasmic antineutrophil cytoplasmic antibody, and perinuclear antineutrophil cytoplasmic antibody, were all normal. On the basis of the findings of these examinations and the patient's history, we diagnosed her as TTS.

Reconstruction of the ala nasi was conducted in a 2-stage fashion. In the first stage, a left paramedian forehead flap and an auricular chondrocutaneous composite graft from the right concha were transferred to cover the surface and inner lining of the alar defect, respectively. Two weeks later, the pedicle of the forehead flap was divided. Her course was uneventful after the second operation. However, 18 months later, she complained of a spread nostril and subsequent xeransis and crust formation on the nostril floor. As a result, she started scratching her nostril floor, and not the reconstructed ala nasi. Therefore, we narrowed the nostril by Z-plasty, and the ulcer healed spontaneously. Subsequently, she stopped scratching, and the ulceration has not recurred for 22 months (Fig 1b).

### Case 2

**Figure 2:**
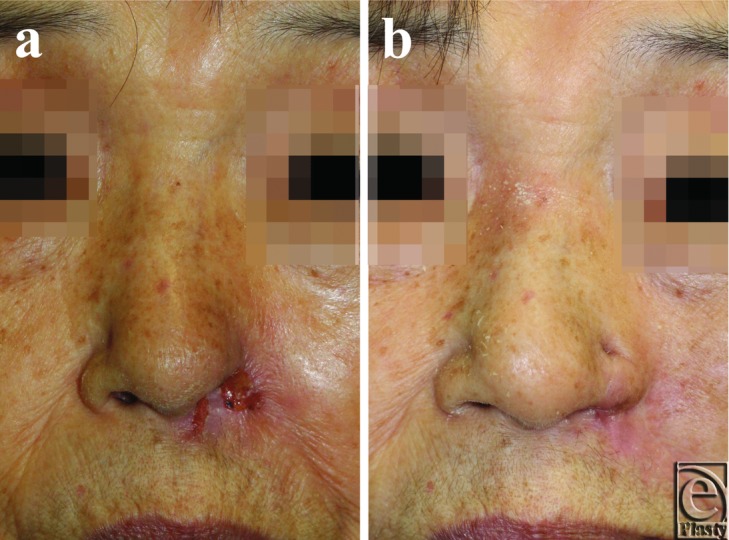
Case 2. (*a*) Preoperative view of the patient at the time of presentation. (*b*) Postoperative view at 5 months. Although the flap was epithelialized by installing the protector at the time of this photograph, she continued to manipulate the reconstructed ala nasi and nasal ulcerations occurred intermittently.

A 79-year-old woman was referred to us with a defect of her ala nasi and ulceration of the left nostril floor (Fig 2a). She had undergone a brain tumor resection 14 years previously, which caused the decreased sensation in her left face. She also developed Alzheimer's dementia 7 years previously. Seven months prior to her visit, her family noticed her manipulation of the nose and a crust formation on her left ala nasi. The lesion slowly progressed to an erosive stage and finally resulted in alar tissue loss. Initially, a biopsy was performed, which showed nonspecific chronic inflammation. The blood test did not show any specific abnormality. On the basis of the patient's history, the appearance and location of the ulceration, and the biopsy result, we diagnosed her as TTS.

We planned a 1-stage reconstruction using a nasolabial flap and an auricular chondrocutaneous composite graft from the concha, because she had dementia. Postoperative wound healing was uneventful, but 10 days after surgery, she started scratching the reconstructed nasal ala. The flap was immediately epithelialized by installing a protector, however, she continued to manipulate the reconstructed ala nasi and nasal ulcerations occurred intermittently (Fig 2b). Although we attempted to treat the ulcer with a protector, she continued to scratch the lesion.

## DISCUSSION

Trigeminal trophic syndrome was first described by Loveman^1^ and McKenzie^2^ in 1933. It presents as a triad of trigeminal sensory impairment, altered sensations of the trigeminal sensory territory, and crescent-shaped ulceration of the nasal ala caused by self-manipulation.^3^ In severe cases, the lesion can involve alar tissue loss and extend to the cheek and upper lip.^4^ The ulcerations of TTS mimic those of other common diseases, including skin neoplasms, vasculitis, infections, and granulomatous disease. Therefore, a biopsy and blood test must be conducted to make a differential diagnosis. When the results of these examinations are negative and patients have a history of damage to the trigeminal nerve system, TTS should be considered as the underlying etiology.^5^

The treatment options reported so far are behavioral modification, pharmaceutical intervention, installation of a protector, transcutaneous nerve stimulation, and surgical repair. Whether innervated flaps or noninnervated flaps should be used for surgical repair remains controversial. Abyholm and Eskeland^6^ reported in 1977 that either local flaps from adjacent parts of the anesthetic area or distant flaps yielded only temporal improvement because they were not innervated. McLean and Watson^7^ successfully performed 1-stage reconstruction using an innervated forehead flap from the healthy side, where the pedicle of the flap was de-epithelialized (not divided) and passed through a subcutaneous tunnel. On the contrary, it was reported recently that a noninnervated local cheek flap from the ipsilateral side,^5^ or a 2- or 3-staged forehead flap, where the pedicle of the flap was divided and the flap became anesthetic,^4^^,^^5^ had healed the ulcerations of TTS. We also used a noninnervated nasolabial flap or a paramedian forehead flap in combination with an auricular chondrocutaneous composite graft. In our case 1, ulceration recurred once on the nostril floor, and not on the reconstructed nasal ala. However, after the patient's discomfort of dry nostril and crust formation was improved by revisional Z-plasty, the ulceration did not recur for 22 months. On the contrary, in our case 2, the patient started scratching her reconstructed ala nasi only 10 days after surgery. Therefore, we could not conclude whether an innervated or noninnervated flap is the optimum procedure, and we must point out that further investigations are needed to determine how innervation of the flap can affect the result of TTS treatment.

## CONCLUSIONS

We reported our experience of 2 patients with TTS treated by surgery. Although TTS is rare, we believe that plastic surgeons should have a raised awareness of this entity and familiarity with the treatment options.
